# Synthesis of novel thiazole, pyranothiazole, thiazolo[4,5-*b*]pyridines and thiazolo[5′,4′:5,6]pyrano[2,3-*d*]pyrimidine derivatives and incorporating isoindoline-1,3-dione group

**DOI:** 10.1186/s13065-019-0559-x

**Published:** 2019-03-26

**Authors:** Mona A. Hosny, Yasser H. Zaki, Wafaa A. Mokbel, Abdou O. Abdelhamid

**Affiliations:** 1 Department of Chemistry, Faculty of Women for Arts, Science and Education, Ain Shams University, Heliopolis, Cairo 11757 Egypt; 2 Department of Chemistry, Faculty of Science, Beni-Suef University, Beni‑Suef, 62514 Egypt; 3 Department of Chemistry, Faculty of Science, Cairo University, Giza, 12613 Egypt

**Keywords:** Aminoacetophenone, Isobenzofuran-1, 3-dione, Thiazole, Hydrazonoyl halide, Thiazolo[4, 5-*b*]pyridine

## Abstract

**Background:**

Thiazole is a core structural motif presents in a wide range of natural products. Thiazole derivatives also have a wide range of medicinal and biological properties.

**Results:**

The reaction of hydrazonoyl halides with 2-(1-(4-(1,3-dioxoisoindolin-2-yl)phenyl)ethylidene)hydrazinecarbothioamidein ethanol and triethylamine yielded 2-(4-(1-(2-(4-(2-Arylhydrazono)-5-s-4,5-dihydrothiazol-2-yl)hydrazono)-ethyl)phenyl)isoindoline-1,3-dione and 2-(4-(1-(2-(5-(2-Arylhydrazono)-4-oxo-4,5-dihydrothiazol-2-yl)hydrazono)ethyl)-phenyl)isoindoline-1,3-dione.The reaction of 2-(4-(1-(2-(4-oxo-4,5-dihydrothiazol-2-yl)hydrazono)ethyl)phenyl)isoindoline-1,3-dione with arylidenemalononitrile also yielded 5-amino-2-(2-(1-(4-(1,3-dioxoisoindolin-2-yl)phenyl)ethylidene)hydrazinyl)-7-substituted-7*H*-pyrano[2,3-*d*]thiazole-6-carbonitrile. The structures of the newly synthesized compound were elucidated whenever possible on the basis of elemental analysis, spectral data, and alternative synthetic routes. Three of them were evaluated against a breast cancer cell line for their antitumor activity.

**Conclusions:**

Compound **(1)** has been shown to be useful in the synthesis of a new series of 1,3-thiazole, pyrano[2,3-*d*]thiazole and 4,5-dihydrothiazolo[4,5-*b*]pyridine derivatives using hydrazonoyl halides as precursors. The anticancer efficacy of compounds **(9b)**, **(9e)**, and **(9f)** against MCF-7, a breast cancer cell line, was also compared to the standard anticancer drug doxorubicin. 
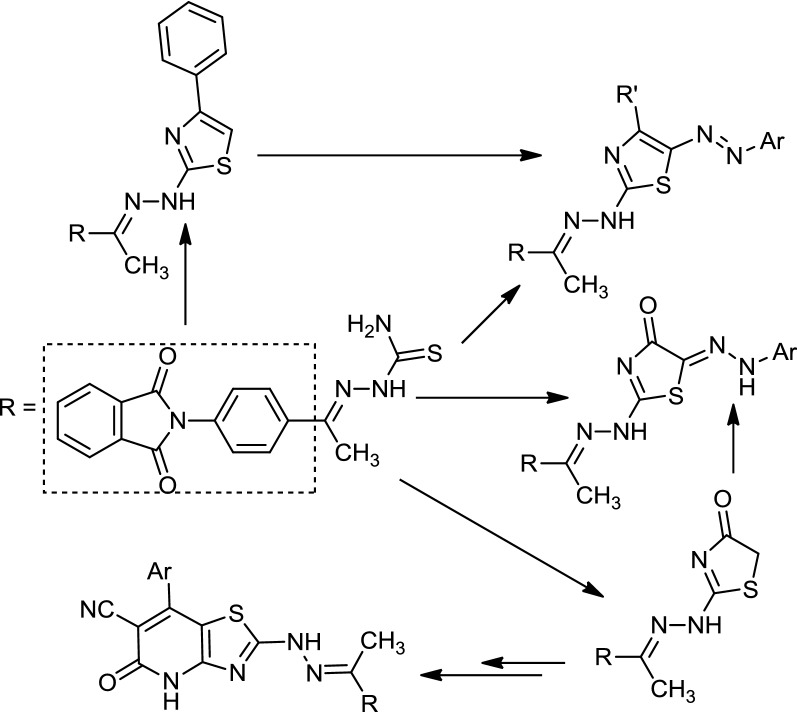

**Electronic supplementary material:**

The online version of this article (10.1186/s13065-019-0559-x) contains supplementary material, which is available to authorized users.

## Introduction

In a variety of natural products, such as vitamin B1 (thiamine) and penicillin, thiazole is a core structural motif. Thiazole derivatives also have a wide range of medicinal and biological properties, including antibacterial, antifungal [1], anti-inflammatory [2], antiviral [3], antimalarial [4], and anti-HIV activities [5]. Thiazole analogs can serve as estrogen receptor ligands [6], neuropeptides [7], and Y5 adenosine receptors [8]. They may inhibit the aggregation factor of human platelets [9], urokinase [10], and poly (ADP-ribose) polymerase-1 [11]. Furthermore, thiazoles are involved in the development of pain therapy drugs [12]. They act as fibrinogenic receptor antagonists with antithrombotic activity [13], and as new bacterial DNA gyrase B inhibitors [14]. Pyrano[2,3-*d*]thiazoles show a wide range of drug development applications against obesity, hyperlipidemia, atherosclerotic diseases [15, 16]. This study is a continuation of our earlier work on the synthesis of biologically active heterocycles [17–21]. The synthesis of new heterocyclic thiazole, pyranothiazole, thiazolo[4,5-b]pyridine and thiazolo[5′,4′:5,6]pyrano[2,3-*d*]pyrimidine derivatives is reported in this document to obtain highly effective antimicrobial and anticancer agents.

## Results and discussion

**Scheme 1:**
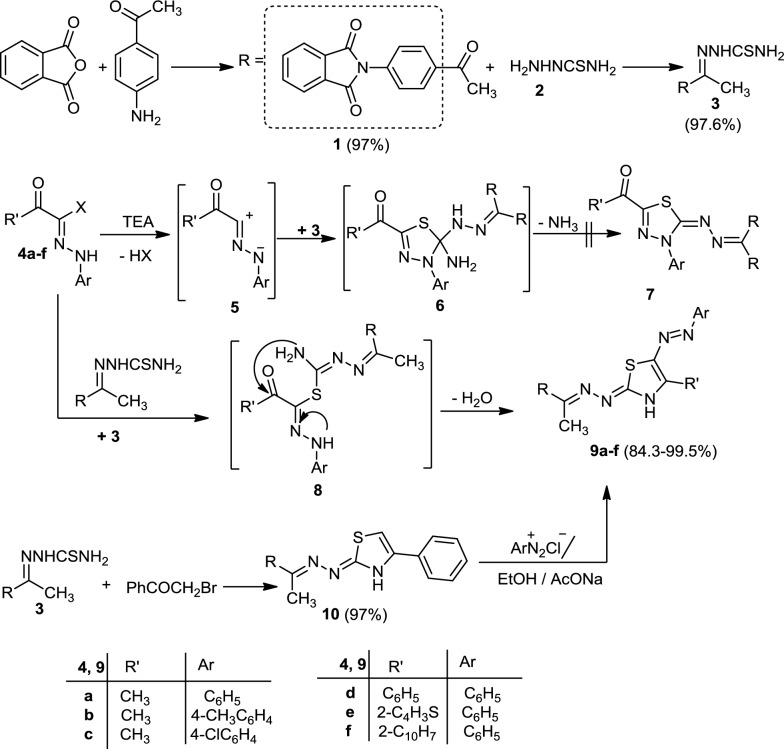
Synthesis of 2-(4-(1-(2-(4-substituted-5-(aryldiazenyl)thiazol-2-yl)hydrazono)ethyl)-phenyl)isoindoline-1,3-dione **(9a–f)**

Treatment of 4-aminoacetophenone with isobenzofuran-1,3-dione in a boiling acetic acid produced 2-(4-acetylphenyl)isoindoline-1,3-dione (**1**). Compound (**1**) was reacted with thiosemicarbazide (**2**) to afford 2-(1-(4-(1,3-dioxoisoindolin-2-yl)phenyl) ethylidene)hydrazinecarbothioamide (**3**). The structure of compound (**3**) was deduced by spectral data, elemental analyses, and chemical transformation. In boiling ethanol containing trimethylamine, compound (**3**) was further reacted with 2-*oxo*-*N*-phenylpropanehydrazonoyl chloride (**4a**) to produce a product that could be isolated by thin layer chromatography (TLC). The compound formula was determined to be: 2-(4-(1-(2-(4-methyl-5-(phenyldiazenyl)thiazol-2-yl)hydrazono)ethyl)phenyl)isoindoline-1,3-dione (**9a**), based on its spectral data and elemental analysis. Similarly, the corresponding hydrazonoyl halides **(4b–f)** were reacted with compound **(3)** to produce 2-(4-(1-(2-(4-substituted 5-(aryldiazenyl)thiazol-2-yl)hydrazono)ethyl)phenyl)isoindoline-1,3-dione **(9b–f)** (Scheme 1). Reaction of compound **(3)** with ω-bromoacetophenone gave 2-(4-(1-(2-(4-phenylthiazol-2-yl)hydrazono)ethyl)phenyl)isoindoline-1,3-dione (**10**). Compound (**10**) has been reacted with benzenediazonium chloride in ethanolic sodium acetate solution to produce a product that is identical in all aspects (mp, mixed mp, and spectra) to compound (**9d**). Considering these results, the mechanism outlined in Scheme 1 seemed to be the most plausible pathway for the formation of **(9)** from the reaction of compound (**4)** or **(5)** with compound (**3)**. The reaction involves the initial thiohydrazonate formation (**8**), which underwent intermolecular cyclization directly to afford **(9)** through elimination of a H_2_O molecule or through 1,3-dipolar cycloaddition of nitrile imine (**5)** to the C=S double bond of **(3)** to afford thiadiazole **(7)** via elimination of NH_3_. Structure (7) was excluded on the basis of elemental analyses, spectra data and alternative synthetic route.

**Scheme 2:**
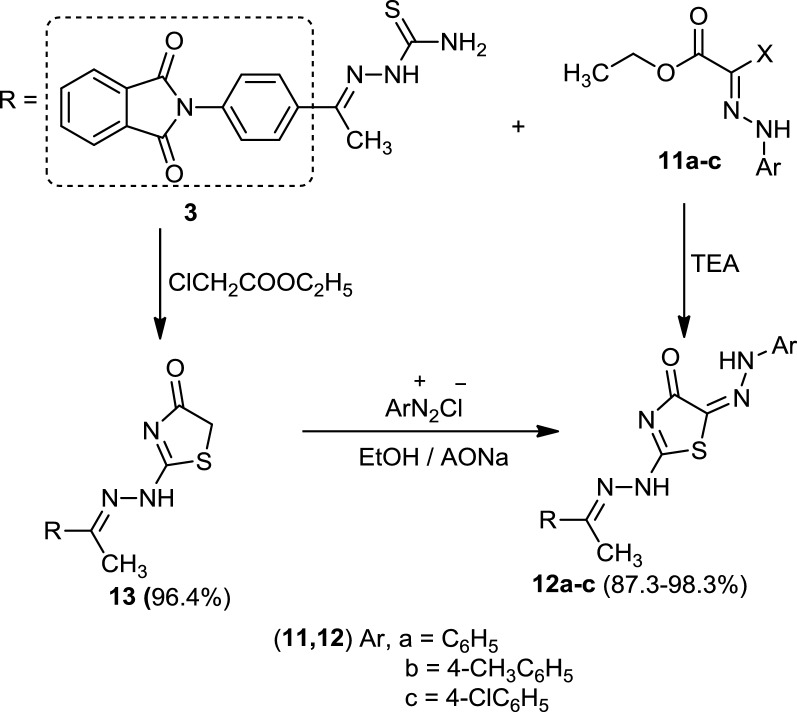
Synthesis of 2-(4-(1-(2-(5-(2-arylhydrazono)-4-oxo-4,5-dihydrothiazol-2-yl)hydrazono)ethyl)phenyl)isoindoline-1,3-dione (**12a–c)**

However, reaction of compound (**3**) with ethyl 2-chloro-2-(2-phenylhydrazono)acetate **(11a)** in ethanol containing a catalytic amount of triethylamine afforded 2-(4-(1-(2-(4-*oxo*-5-(2-phenylhydrazono)-4,5-dihydrothiazol-2-yl)hydrazono)ethyl)phenyl)-isoindoline-1,3-dione (**12a**). The spectral data, elemental analyses, and alternative syntheses elucidated the structure of compound **(12a)**. Reaction of ethyl chloroacetate with compound **(3)** in boiling ethanol yielded 2-(4-(1-(2-(4-oxo-4,5-dihydrothiazol-2-yl)hydrazono)ethyl)phenyl)-isoindoline-1,3-dione (**13**). Coupling of benzenediazonium chloride with (**13**) in a cold solution of ethanolic sodium acetate gave a product identical in all aspects (mp, mixed mp, and spectra) with compound **(12a)** (Scheme 2). Analogously, reaction of hydrazonoyl chlorides **(11b)** and **(11c)** with compound **(3)** in ethanolic triethylamine afforded 2-(4-(1-(2-(4-oxo-5-(2-(*p*-tolyl)hydrazono)-4,5-dihydrothiazol-2-yl)hydrazono)-ethyl)phenyl)isoindoline-1,3-dione (**12b**) and 2-(4-(1-(2-(5-(2-(4-chlorophenyl)hydrazono)-4-oxo-4,5-dihydrothiazol-2-yl)-hydrazono)ethyl)phenyl)isoindoline-1,3-dione (**12c**) (Scheme 2).

**Scheme 3:**
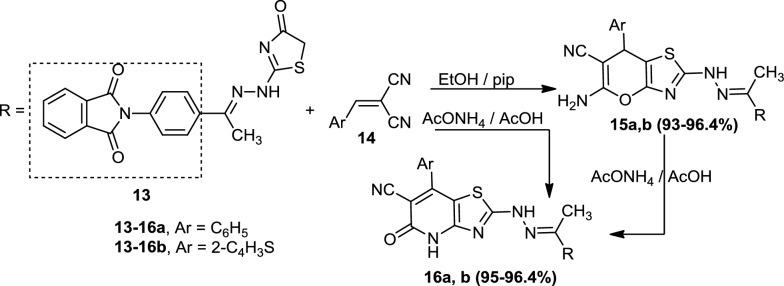
Synthesis of 5-Amino-2-(2-(1-(4-(1,3-dioxoisoindolin-2-yl)phenyl)ethylidene)-hydrazinyl)-7-substituted-7*H*-pyrano[2,3-*d*]thiazole-6-carbonitrile **(15a, b)** and 2-(2-(1-(4-(1,3-dioxoisoindolin-2-yl)phenyl)ethylidene)hydrazinyl)-5-oxo-7-substituted-4,5-dihydrothiazolo[4,5-*b*]pyridine-6-carbonitrile **(16a, b)**

Ultimately, the treatment of 2-(4-(1-(2-(4-oxo-4,5-dihydrothiazol-2-yl)hydrazono)ethyl)phenyl)-isoindoline-1,3-dione (**13**) with the appropriate 2-(arylemethylene)malononitrile **(14a)** and (**14b)** in ethanol containing a catalytic amount of piperidine afforded 5-amino-2-(2-(1-(4-(1,3-dioxoisoindolin-2-yl)phenyl)ethylidene)-hydrazinyl)-7-phenyl-7*H*-pyrano[2,3-*d*]thiazole-6-carbonitrile (**15a)** and 5-amino-2-(2-(1-(4-(1,3-dioxoisoindolin-2-yl)phenyl)ethylidene)hydrazinyl)-7-(thien-2-yl)-7*H*-pyrano[2,3-*d*]thiazole-6-carbonitrile **(15b),** respectivily. The corresponding structures of these compounds were elucidated by spectral data, elemental analyses, and chemical transformation. Boiling compounds (**15a)** and (**15b)** in acetic acid and ammonium acetate afforded 2-(2-(1-(4-(1,3-dioxoisoindolin-2-yl)phenyl)ethylidene)hydrazinyl)-5-oxo-7-phenyl-4,5-dihydrothiazolo[4,5-*b*]pyridine-6-carbonitrile (**16a**) and 2-(2-(1-(4-(1,3-dioxoisoindolin-2-yl)phenyl)ethylidene)hydrazinyl)-5-oxo-7-(thien-2-yl)-4,5-dihydrothiazolo[4,5-*b*]pyridine-6-carbonitrile **(16b**), respectively (Scheme 3). The previous reaction was carried out in acetic acid containing ammonium acetate to afford products identical in all aspects (mp, mixed mp, and spectra) with **(16a)** and (**16b)** (Scheme 3).

### Cytotoxicity evaluations

**Fig. 1:**
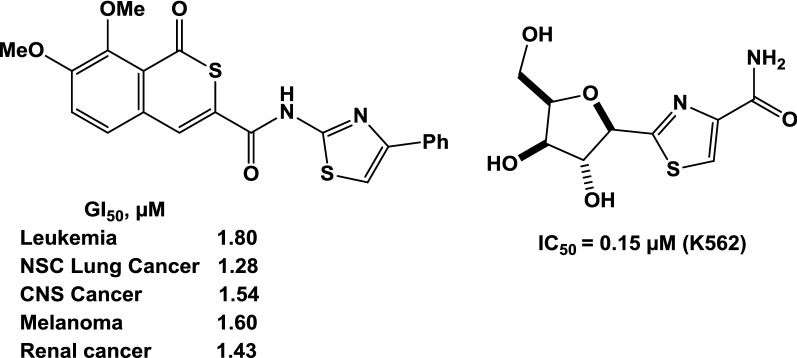
Antitumor activity of thiazoles

Through the literary survey it became clear to us that many thiazole derivatives have an excellent anti-tumor activity as shown in Fig. 1 [22, 23]. In light of this, anti-tumor activity was examined for a new series of thiazole substitutes against breast cancer cells line (MCF-7).

**Fig. 2:**
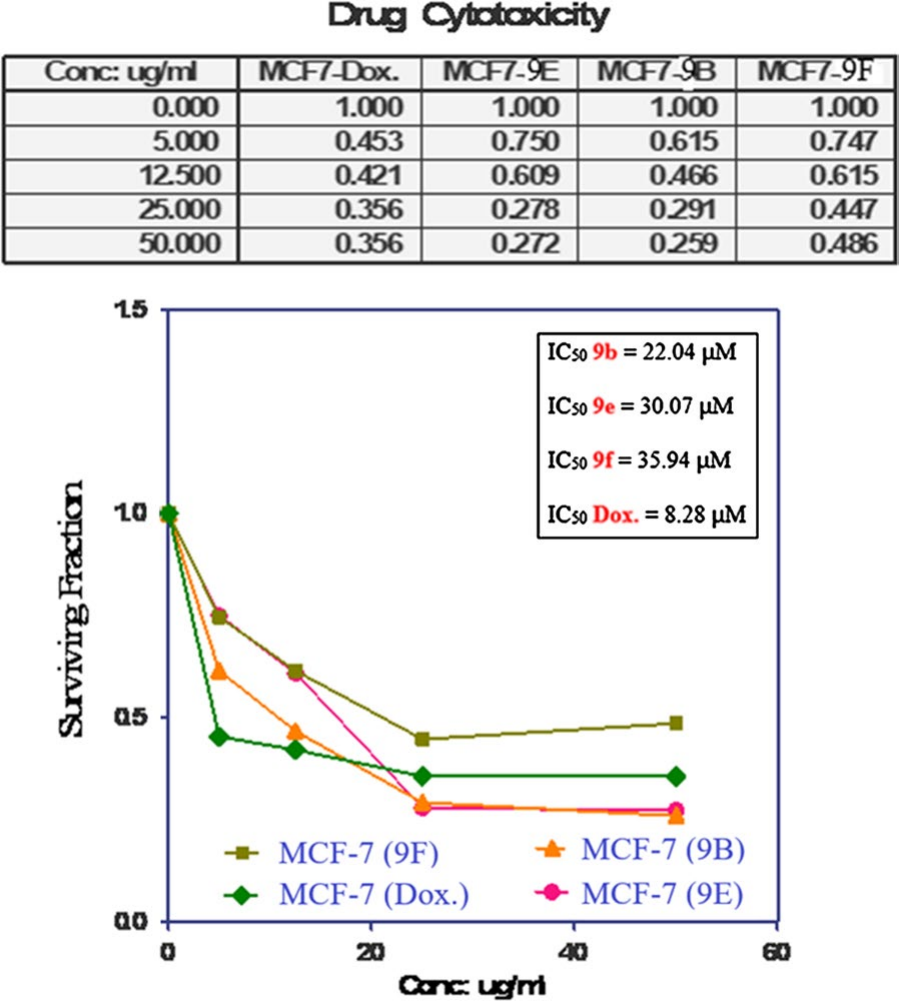
Cytotoxicity (IC_50_, μM) of the synthesized compounds (**9b), (9e)** and **(9f**) against the MCF-7 breast cell line

In comparison with the well—known anticancer standard drug doxorubicin, the in vitro growth inhibitory activity of the synthesized compounds (**9b**), (**9e**), and (**9f**) was investigated using trypan blue dye viability test. Data generated were used to determine a dose response curve that determined the concentration of test compounds needed to kill 50% of the cell population (IC_50_). The cytotoxic activity of three independent experiments was expressed as the mean IC_50_. In a concentration-based manner, the results showed that all tested compounds showed an inhibitory activity for tumor cell lines. The small IC_50_ values for the compounds selected indicate that, higher concentrations may be used for more anticancer effect. The results are shown in Table 1 and Fig. 2 as follows:

**Table 1:** The in vitro inhibitory activity of tested compounds against breast cancer cell line expressed as IC_50_ values (μM)

Compound no.	MCF-7 IC_50_ (µM)
Doxorubicin	8.28
**9b**	22.04
**9e**	30.07
**9f**	35.94

The in vitro inhibitory activities of tested compounds against breast cancer cell line (MCF-7) have the following descending order: **(9b)** > (**9e)** > (**9f)**.

Examination of the SAR leads to the following conclusions:

- For substituents in position 4 and 5 of the thiazole ring, the following descending order is the in vitro inhibitory activity of tested compounds against the breast cancer cell line. The activity of thiazole (**9e**) is moderate.
- For substituents at position 4 and 5 of the thiazole ring, the in vitro inhibitory activity of tested compounds against breast cancer cell line has the following descending order: CH_3_, 4-CH_3_C_6_H_4_ > 2-C_4_H_3_S, C_6_H_5_ > 2-C_10_H_7_, C_6_H_5_ group.

### Experimental

All of the melting points were determined using a Gallenkamp electrothermal melting point apparatus (Laim George, Calgary, AB, Canada) and, they are uncorrected. The IR (cm^−1^) spectra were recorded using a KBr disk on a FTIR-8201 spectrophotometer (Shimadzu, Tokyo, Japan). The ^1^H NMR and^13^C NMR spectra were recorded in DMSO-d6 on a Bruker Bio Spin AG spectrometer (Bruker, Switzerland) at 400 and 100 MHz. Mass spectra were recorded at 70 eV on a Shimadzu GCMS-QP1000 EX mass spectrometer (Tokyo, Japan). Elemental analyses were conducted at the Microanalytical Center of Cairo University. All reactions were followed by *TLC* (silica gel, Merck, Kenilworth, NJ, USA). As reported, hydrazonoyl halides have been prepared [24–29].

### Synthesis of 2-(4-Acetylphenyl)isoindoline-1,3-dione (1)

In 10 mL acetic acid, a mixture of 4-aminoacetophenone (1.35 g, 10 mmol) and isobenzofuran-1,3-dione (1.48 g, 10 mmol) was heated for 2 h under reflux. The solid was gathered and crystallized from ethanol, with a yield of 2.54 g (97%), mp: 230–232 °C; IR (KBr, cm^−1^): 3087 (C–H aromatic), 2963, 2893 (C–H), 1706 (C=O), 1617 (C=C);^1^H-NMR (CDCl_3_)δ: 2.49 (s, 3H, CH_3_), 6.70–6.73 (d, 2H, *J *= 8 Hz, Ar–H), 7.99–8.00 (d, 2H, *J* = 8 Hz, Ar–H),8.01–8.03 (d, 2H, *J* = 8 Hz, Ar–H), 8.17–8.19 (d, 2H, *J* = 8 Hz, Ar–H); ^13^C-NMR (100 MHz) (DMSO-*d*6) δ: 26.2,117.4,125.9, 129.6, 130.1, 131.5, 33.3, 134.6, 165.4, 196.3. *Anal.* Calcd. for C_16_H_11_NO_3_ (265.26): C, 72.45; H, 4.18; N, 5.28; found: C, 72.54; H, 4.21; N, 5.37.

### Synthesis of 2-(1-(4-(1,3-dioxoisoindolin-2-yl)phenyl)ethylidene)-hydrazinecarbothioamide (3) Additional file 1: Figure S1

A mixture of 2-(4-acetylphenyl)isoindoline-1,3-dione (**1**) (2.65 g, 10 mmol) and thiosemicarbazide (0.97 g, 10 mmol) in 20 mL ethanol, as well as a few drops of concentrated HCl, was heated for 30 min under reflux. The bright beige needles were gathered and recrystallized from ethanol, with a yield of 2.9 g (97.6%), mp: 250 °C; IR (KBr, cm^−1^): 3319, 3262, 3151 (NH, NH_2_), 1706 (CO),1617 (C=N),1174 (C=S); ^1^H-NMR(CDCl_3_)δ: 2.35 (s, 3H, CH_3_), 7.47 (d, 2H, *J* = 8 Hz, Ar–H), 7.66 (d, 2H, *J* = 8 Hz, Ar–H), 7.83(d, 2H, *J* = 8 Hz, Ar–H), 7.93–8.06 (m, 4H, Ar–H), 8.29 (s, br. 1H, NH); ^13^C-NMR (100 MHz) (DMSO-d_6_) δ: 18.9, 123.9, 127.3, 127.5, 131.9, 133.1, 135.3, 137.7, 147.8, 167.4, 179.6. MS; m/z %: 338 (M^+^, 4.3), 324 (17.6), 323 (86), 321 (44), 304 (15), 278 (24), 264 (35), 263 (83), 249 (46), 222 (44), 204 (17), 166 (27), 139 (14), 116 (17), 104 (57), 102 (24), 90 (22), 77 (30), 76 (100), 59 (17), 50 (24). *Anal.* Calcd. for C_17_H_14_N_4_O_2_S (338.38):C, 60.34; H, 4.17; N, 16.56; S, 9.48; found: C, 60.22; H, 4.14; N, 16.68; S, 9.52.

### Synthesis of 2-(4-(1-(2-(4-(2-Arylhydrazono)-5-s-4,5-dihydrothiazol-2-yl)hydrazono)ethyl)phenyl)isoindoline-1,3-dione (**9a–f**)

A mixture of the appropriate hydrazonoyl halides (**4a–f)** (1 mmol), 2-(1-(4-(1,3-dioxoisoindolin-2-yl)phenyl) ethylidene)hydrazinecarbothioamide(**3**) (0.338 g, 1 mmol) in ethanol (20 mL) and triethylamine (0.15 mL, 1 mmol) was heated for 2 h under reflux. The formed solid in this way was gathered and crystallized from acetic acid. Products **9a–f** were prepared together with their physical constants, and they are described as follow.

### 2-(4-(1-(2-(4-Methyl-5-(phenyldiazenyl)thiazol-2-yl)hydrazono)ethyl)phenyl)-isoindoline-1,3-dione (**9a**) Additional file 2: Figure S2

Scarlet-red (98.3% yield); mp: 240 °C; IR (KBr, cm^−1^): 3326 (NH), 1710 (CO),1609 (C=N),1492 (N=N); ^1^H-NMR (CDCl_3_) δ:2.19 (s, 3H, CH_3_), 2.35 (s, 3H, CH_3_), 7.33–7.27 (m, 5H, Ar–H), 7.54 (d, 2H, *J *= 8 Hz, Ar–H), 7.81 (d, 2H, *J *= 8 Hz, Ar–H), 7.79 (d, 2H, *J *= 8 Hz, Ar–H), 8.07 (d, 2H, *J *= 8 Hz, Ar–H), 8.11 (s, br., 1H, NH); ^13^C-NMR (100 MHz) (DMSO-d_6_) δ: 15.7, 18.9, 114.8, 122.8, 123.9, 127.3, 127.5, 127.8, 129.7, 131.9, 133.1, 135.3, 127.7, 143.9, 147.9, 167.4, 179.6. MS; m/z %: 481(M^+^, 33), 480 (97), 479 (32), 465 (35),447 (64), 339 (21), 375 (24),343 (16), 288 (17),275 (13),263 (77), 249 (35), 222 (48), 204 (19),166 (39),140 (16),105 (18, 93 (19), 77 (100), 76 (65), 51(15). *Anal.* Calcd. For C_26_H_20_N_6_O_2_S (480.54):C, 64.98; H, 4.20; N, 17.49; S, 6.67; found: C, 65.10; H, 4.32; N, 17.52; S, 6.79.

### 2-(4-(1-(2-(4-Methyl-5-(*p*-tolyldiazenyl)thiazol-2-yl)hydrazono)ethyl)phenyl)-isoindoline-1,3-dione (**9b**) Additional file 3: Figure S3

Orange (84.3% yield); mp: 170 °C; IR (KBr,cm^−1^): 3269 (NH), 1710 (C=O),1598 (C=N); ^1^H-NMR(CDCl_3_) δ:2.19 (s, 3H, CH_3_), 2.34 (s, 6H, 2CH_3_), 7.33 (d, 2H, *J* = 8 Hz, Ar–H), 7.64 (d, 2H, *J* = 8 Hz, Ar–H), 7.93 (d, 2H, *J* = 8 Hz, Ar–H), 7.96–8.01 (m, 6H, Ar–H), 8.89 (s, br., 1H, NH); ^13^C-NMR (100 MHz) (DMSO-d_6_) δ: 14.4, 25.8, 116.9, 123.9, 127.4, 127.5, 129.6, 131.9, 135.3, 147.8, 167.4. MS; m/z %: 494 (M^+^,0.3), 338 (16), 323 (32), 321 (28), 264 (20), 263 (55), 249 (25), 222 (33), 204, (12), 166 (21), 130 (15), 104 (46), 102 (20), 90 (20),77 (28), 76 (100), 63 (14), 50 (25). *Anal.* Calcd. For C_27_H_22_N_6_O_2_S (494.57): C, 65.57; H, 4.48; N, 16.99; S, 6.48; found: C, 65.46; H, 4.55; N, 17.09; S, 6.58.

### 2-(4-(1-(2-(5-((4-Chlorophenyl)diazenyl)-4-methylthiazol-2-yl)hydrazono)ethyl)-phenyl)isoindoline-1,3-dione (***9*c**) Additional file 4: Figure S4

Dark orange (98.5% yield);mp: 210–240 °C; IR (KBr, cm^−1^): 3279 (NH), 1709 (C=O), 1595(C=N); ^1^H-NMR(CDCl_3_) δ:2.39 (s, 3H, CH_3_), 2.56 (s, 3H, CH_3_), 7.52 (d, 2H, *J* = 8 Hz, Ar–H), 7.56 (d, 2H, *J* = 8 Hz, Ar–H), 7.80 (d, 2H, *J* = 8 Hz, Ar–H), 7.90 (d, 2H, *J* = 8 Hz, Ar–H), 7.92–8.5 (m, 4H, Ar–H), 8.89 (s, br., 1H, NH); ^13^C-NMR (100 MHz) (DMSO-d_6_) δ: 9.0, 14.3, 123,9, 127.3, 127.5, 130.2, 131.9, 133.1, 135.3, 137.7, 147.9, 167.4, 179.6. MS (m/z  %): 516 (M^+2^, 11), 514(M^+^, 27), 247 (17), 263 (31), 249 (24), 222 (22), 166 (25), 140 (13), 139 (17), 111(41), 104 (44), 102 (29), 90 (23), 77 (30)), 76 (100), 50 (30). *Anal.* Calcd. For C_26_H_19_ClN_6_O_2_S (514.99): C, 60.64; H, 3.72; N, 16.32; S, 6.23.; Found: C, 60.82; H, 3.57; N, 16.12; S, 6.45.

### 2-(4-(1-(2-(4-Phenyl-5-(phenyldiazenyl)thiazol-2-yl)hydrazono)ethyl)phenyl)-isoindoline-1,3-dione (***9*d**) Additional file 5: Figure S5

Red needles (99.5% yield); mp: 240 °C; IR (KBr, cm^−1^): 3065 (NH), 1709 (C=O), 1598 (C=N); ^1^H-NMR (CDCl_3_) δ: 2.38 (s, 3H, CH_3_), 7.03–8.29 (m, 18 H, Ar–H) and 10.12 (s, 1H, NH). MS (m/z %): 542 (M^+^, 45), 514 (20), 438 (24), 263 (49), 249 (32), 222 (29), 166 (27), 105 (18%), 104 (48%), 77 (100), 76 (56), 51(15). *Anal*. Calcd. For C_31_H_22_N_6_O_2_S (542.61) C, 68.62; H, 4.09; N, 15.49; S, 5.91; Found: C, 68.55; H, 4.15; N, 15.56; S, 6.11.

### 2-(4-(1-(2-(5-(Phenyldiazenyl)-4-(thien-2-yl)thiazol-2-yl)hydrazono)ethyl)phenyl)-isoindoline-1,3-dione (***9*e**) Additional file 6: Figure S6

Yellow needles (86% yield); mp: 230 °C; IR (KBr, cm^−1^): 3276 (NH), 1707 (C=O), 1609 (C=N); ^1^H-NMR(CDCl_3_) δ: 2.38 (s, 3H, CH_3_), 6.82 (s, 1H, thienyl-H4), 7.02–7.17 (m, 2H, thienyl H3, H5), 7.37 (d, 2H, *J* = 8 Hz, Ar–H), 7.52 (d, 2H, *J* = 8 Hz, Ar–H), 7.80 (d, 2H, *J* = 8 Hz, Ar–H), 7.97–8.01 (m, 7H, Ar–H), 10.14 (s, 1H, NH); ^13^C-NMR (100 MHz) (DMSO-d_6_) δ: 14.3, 91.4, 112.7, 112.9, 117.5, 121.2, 122.3, 123.9, 126.4, 127.4, 127.5, 128.7, 129.3, 129.5, 129.6, 130.0, 131.8, 133.0, 134.0, 135.3, 137.6, 143.4, 148.0, 149.6, 151.1, 152.7, 160.8, 167.0, 167.4, 179.4. MS; m/z %: 550 (M+2, 6.8), 338 (47), 323 (100), 321 (48), 278 (26), 264 (31), 263 (75), 249 (31), 222 (33), 166 (19), 130 (14), 116 (12), 104 (33), 102 (15) 77 (22), 76 (62), 57(19). *A*nal. Cald. For C_29_H_20_N_6_O_2_S_2_ (548.64) C, 63.49; H, 3.67; N, 15.32; S, 11.69; Found: C, 63.55; H, 3.78; N, 15.29; S, 11.74.

### 2-(4-(1-(2-(4-(Naphthalen-2-yl)-5-(phenyldiazenyl)thiazol-2-yl)hydrazono)ethyl)-phenyl)isoindoline-1,3-dione (***9*f**) Additional file 7: Figure S7

Red needles (99.5% yield); mp: 248 °C; IR (KBr, cm^−1^): 3060 (NH), 1708 (CO), 1601 (C=N), 1547(N=N). ^1^H-NMR (CDCl_3_) δ: 2.38 (s, 3H, CH_3_), 7.40–7.50 (m, 3H, Ar–H), 7.54 (d, 2H, *J* = 8 Hz, Ar–H), 7.92–8.12 (m, 12H, Ar–H), 8.16 (d, 2H, *J* = 8 Hz, Ar–H), 8.76 (s, 1H, Ar–H), 9.74 (s, br., 1H, NH). *A*nal. Cald. For C_35_H_24_N_6_O_2_S (592.67) C, 70.93; H, 4.08; N, 14.18; S, 5.41; Found: C, 71.12; H, 4.15; N, 14.22; S, 5.60.

### Synthesis of 2-(4-(1-(2-(4-phenylthiazol-2-yl)hydrazono)ethyl)phenyl)-isoindoline-1,3-dione (10) Additional file 8: Figure S8

In 10 mL ethanol, a mixture of compound (**1**) (0.26 g, 10 mmol) and phenacylbromide (1.99 g, 10 mmol) was heated for 2 h under reflux. A yellow ppt. has been gathered and recrystallized from methanol (97% yield); mp: 260–262 °C; IR (KBr,cm^−1^): 3067 (NH), 1714 (C=O), 1611 (C=N), 1508 (C=C); ^1^H-NMR (CDCl_3_)δ: 2.38 (s, 3H, CH_3_), 7.28 (s, 1H, thiazole H–5), 7.30 (d, 2H, *J *= 8 Hz, Ar–H), 7.50 (d, 2H, *J *= 8 Hz, Ar–H), 7.86–7.97 (m, 9H, Ar–H and (s, 1H, NH); ^13^C-NMR (100 MHz) (DMSO-d_6_) δ: 14.5, 104.6, 123.9, 126.1, 126.6, 127.5, 128.0, 129.1, 131.9, 132.6, 135.1, 135.3, 138.1, 146.7, 150.7, 167.4, 170.2. MS (m/z  %): 439 (M + 1, 31), 438 (100), 263 (36), 249 (14), 222 (11). *A*nal. Calcd. for C_25_H_18_N_4_O_2_S (438.50): C, 68.48; H, 4.14; N, 12.78; S, 7.31, Found: C, 68.55; H, 4.27; N, 12.88; S, 7.42.

### Synthesis of 2-(4-(1-(2-(5-(2-arylhydrazono)-4-*oxo*-4,5-dihydrothiazol-2-yl)hydrazono)ethyl)-phenyl)isoindoline-1,3-diones (**12a–c**)

In an ice bath, a mixture of compound (**13**) (0.38 g, 1 mmol) and sodium acetate trihydrate (0.138 g, 1 mmol) cooled to 0–5 °C in 20 mL ethanol. Appropriate quantities of arenediazonium chloride [prepared by diazotizing replaced amines (1 mmol) dissolved in hydrochloric acid (0.3 mL, 6 M) with sodium nitrite solution (0.07 g, 1 mmol) in H_2_O (2 mL)] were added in portion to the previous mixture while stirring.. After complete addition, the reaction mixture was stirred in an ice bath for another 2 h. The yellow solid has been filtered off, washed with H_2_O, and finally recrystallized from ethanol, to give **(12a–c)**.

### 2-(4-(1-(2-(4-*Oxo*-5-(2-phenylhydrazono)-4,5-dihydrothiazol-2-yl)hydrazono)ethyl)-phenyl)isoindoline-1,3-dione (***12*a**) Additional file 9: Figure S9

Yellow solid (98.3% yield); mp: 230–232 °C; IR (KBr, cm^−1^): 3279 (NH), 1708 (CO), 1595 (C=N); ^1^H-NMR (CDCl_3_) δ:2.35 (s, 3H, CH_3_), 7.47–7.49 (m, 3H, Ar–H), 7.90–8.06 (m, 10H, Ar–H), 10.31 (s, br., 1H, NH), 10.54 (s, br., 1H, NH); ^13^C-NMR (100 MHz) (DMSO-d_6_) δ: 14.3, 123.9, 127.3, 127.5, 131.9, 133.1, 135.3, 137.7, 137.7, 167.4, 179.6. MS; m/z %: 482 (M^+^, 1.6), 338 (57), 323 (100), 321 (80), 278 (24), 263 (93), 249 (20), 222 (30), 104 (14), 76 (40), 50 (12). *A*nal. Calcd. For C_25_H_18_N_6_O_3_S (482.51) C, 62.23; H, 3.76; N, 17.42; S, 6.65; Found: C, 62.12; H, 3.62; N, 17.29; S, 6.58.

### 2-(4-(1-(2-(4-*Oxo*-5-(2-(*p*-tolyl)hydrazono)-4,5-dihydrothiazol-2-yl)hydrazono)-ethyl)phenyl)isoindoline-1,3-dione (**12b**) Additional file 10: Figure S10

Dark yellow needles, recrystallized from methanol (98.3% yield); mp: 210–250 °C; IR (KBr, cm^−1^): 3276 (NH), 1709 (CO), 1597 (C=N); ^1^H-NMR (CDCl_3_) δ:2.27 (s, 3H, CH_3_), 2.34 (s, 3 H, CH_3_),7.47 (d, 2H, *J* = 8 Hz, Ar–H), 7.62 (d, 2H, *J* = 8 Hz, Ar–H), 7.91–8.11 (m, 8H, Ar–H), 10.28 (s, br., 1H, NH), 10.56 (s, br., 1H, NH); ^13^C-NMR (100 MHz) (DMSO-d6) δ: 14.4, 27.2, 123.9, 124.0, 127.4, 127.5, 129.2, 131.9, 133.1, 135.3, 135.4, 136.4, 147.8, 167.4, 179.6. MS; m/z %: 496 (M^+^, 0.43), 338 (17), 323 (37), 321 (48), 278 (12), 263 (89), 249 (41), 222 (48), 166 (30, 130 (16), 105 (17), 104 (58), 90 (19), 77 (27), 76 (100), 50 (21). *A*nal. Calcd. For C_26_H_20_N_6_O_3_S (496.54) C, 62.89; H, 4.06; N, 16.93; S, 6.46; Found: C, 62.77; H, 4.12; N, 17.11; S, 6.35.

### 2-(4-(1-(2-(5-(2-(4-Chlorophenyl)hydrazono)-4-oxo-4,5-dihydrothiazol-2-yl)-hydrazono)ethyl)phenyl)isoindoline-1,3-dione (**12c**) Additional file 11: Figure S11

Orange (87.3% yield), mp: 288 °C.; IR (KBr, cm^−1^): 3276 (NH), 1709 (CO), 1597 (C=N); ^1^H-NMR (CDCl_3_) δ:2.34 (s, 3 H, CH_3_), 7.47 (d, 2H, *J* = 8 Hz, Ar–H), 7.62 (d, 2H, *J* = 8 Hz, Ar–H), 7.91–8.11 (m, 8H, Ar–H), 10.17 (s, br., 1H, NH), 10.52 (s, br., 1H, NH). *Anal.* Calcd. For C_25_H_17_ClN_6_O_3_S (516.96) C, 58.08; H, 3.31; N, 16.26; S, 6.20 Found: C, 58.12; H, 3.15; N, 16.34; S, 6.05.

### Synthesis of 2-(4-(1-(2-(4-oxo-4,5-dihydrothiazol-2-yl)hydrazono)ethyl)-phenyl)isoindoline-1,3-dione (**13**) Additional file 12: Figure S12

In 20 mL ethanol, a mixture of ethyl chloroacetate (0,12 g, 1 mmol) and compound (**3**) (0,338 g, 1 mmol) was heated for 2 h under reflux to provide a solid recrystallized from ethanol to give a bright yellowish needle (96.4%, yield), mp: 258 °C; IR (KBr,cm^−1^):3265 (NH), 1706 (CO), 1617 (C=N); ^1^H-NMR(CDCl_3_) δ:2.35 (s, 3H, CH_3_), 3.9 (s, 2H, CH_2_), 7.47–7.49 (m, 2H, Ar–H), 7.90–8.06 (m, 6H, Ar–H), 9.36 (s, br., 1H, NH); ^13^C-NMR (100 MHz) (DMSO-d_6_) δ: 14.3, 38.9, 115.9, 125.8, 128.5, 131.8, 134.7, 137.3, 139.7, 150.9, 167.4, 169.3, 182.7. MS (m/z %): 378 (M^+^, 0.76%), 338 (57.52%), 323 (100%), 321 (47.62%), 278 (27.75%), 264 (27.6%), 263 (68.59%), 249 (34.8%), 222 (22.87%), 104 (12.22%), 76 (28.68%), 249 (41.21%), 222 (48.26%), 166 (30.74%), 130 (16.7%), 105 (17.94%), 104 (58.09%), 90 (19.01%), 77 (27,71%), 76 (100%), 50(21.59%). *Anal.* Calcd. For C_19_H_14_N_4_O_3_S (378.40) C, 60.31; H, 3.73; N, 14.81; S, 8.47 Found: C, 60.12; H, 3.85; N, 14.94; S, 8.62.

### Synthesis of 5-amino-2-(2-(1-(4-(1,3-dioxoisoindolin-2-yl)phenyl)ethylidene)-hydrazinyl)-7-substituted-7*H*-pyrano[2,3-*d*]thiazole-6-carbonitrile (**15a**) and (**15b**)

A mixture of compound (**13**) (0.365 g, 1 mmol) and the corresponding arylidenemalonitrile (1 mmol) in 20 mL ethanol containing a catalytic amount of piperidine was heated for 2 h under reflux. The solid was gathered from ethanol and crystallized to produce compounds (**15a**) and (**15b**).

### 5-Amino-2-(2-(1-(4-(1,3-dioxoisoindolin-2-yl)phenyl)ethylidene)hydrazinyl)-7-phenyl-7H-pyrano[2,3-*d*]thiazole-6-carbonitrile (15a) Additional file 13: Figure S13

White crystal (96.4%, yield); mp: 265 °C; IR (KBr, cm^−1^): 3399, 3320, 3263(NH, NH_2_), 2201 (CN), 1705 (C=O),1617 (C=N).^1^H- NMR(CDCl_3_) δ: 2.35 (s, 3H, CH_3_), 4.34 (s, 1H, pyran H–4), 7.47–7.49 (m, 3H, Ar–H), 7.5 (d, 2H, *J* = 8 Hz, Ar–H), 7.8 (d, 2H, *J* = 8 Hz, Ar–H), 7.9 (d, 2H, *J* = 8 Hz, Ar–H), 7.95–8.05 (m, 7H, Ar–H, NH, NH_2_); ^13^C-NMR (100 MHz) (DMSO-d_6_): δ: 18.8, 56.6, 123.0, 127.3, 127.5, 131.9, 133.1, 135.2, 137.7, 147.8, 167.4, 179.6. *Anal*. Calcd. For C_29_H_20_N_6_O_3_S (532.58) C, 65.40; H, 3.79; N, 15.78; S, 6.02 Found: C, 65.14; H, 3.82; N, 15.85; S, 5.91.

### 5-Amino-2-(2-(1-(4-(1,3-dioxoisoindolin-2-yl)phenyl)ethylidene)hydrazinyl)-7-(thien-2-yl)-7H-pyrano[2,3-d]thiazole-6-carbonitrile (15b) Additional file 14: Figure S14

Black crystals (93%, yield);mp: 112–114 °C; IR (KBr, cm^−1^): 3399, 3320 (NH_2_), 3265 (NH), 2201 (CN), 1706 (C=O), 1615 (C=N).^1^H-NMR (CDCl_3_) δ: 2.39 (s, 3H, CH_3_), 4.60 (s, 1H, pyran H–4), 7.51–7.53 (d, 2H, *J* = 8 Hz, Ar–H), 7.65–7.82 (m, 8H, Ar–H, NH, NH_2_), 8.01–8.5 (m, 4H, Ar–H); ^13^C-NMR (100 MHz) (DMSO-d_6_): δ14.3, 18.8, 23.6, 123.9, 127.4, 127.5, 131.9, 133.0, 135.3, 137.6, 148.0, 167.5, 179.4. MS (m/z %): 538 (M^+^, 1.7), 511 (17), 472 (10), 328 (52), 323 (100), 322 (20), 260 (100), 249 (15), 222 (13), 104 (27), 76 (100). *Anal.* Calcd. For C_27_H_18_N_6_O_3_S_2_ (538.60) C, 60.21; H, 3.37; N, 15.60; O, 8.91; S, 11.91 Found: C, 60.02; H, 3.25; N, 15.84; S, 12.10.

### Synthesis of 7-aryl-2-(2-(1-(4-(1,3-dioxoisoindolin-2-yl)phenyl)ethylidene)-hydrazinyl)-5-oxo-4,5-dihydrothiazolo[4,5-*b*]pyridine-6-carbonitrile (**16a**) and (**16b**)

A mixture of the appropriate compound (**15a**) or (**15b**) and ammonium acetate (0.53 g, 1 mmol) was heated for 2 h in acetic acid (15 mL) under reflux. To obtain (**16a**) and (**16b**), the solid was collected and recrystallized from methanol.

### 2-(2-(1-(4-(1,3-Dioxoisoindolin-2-yl)phenyl)ethylidene)hydrazinyl)-5-oxo-7-phenyl-4,5-dihydrothiazolo[4,5-b]pyridine-6-carbonitrile (**16a**)

Beige solid needles (96.4%, yield); mp: 265 °C; IR (KBr, cm^−1^): 3265 (NH), 2200 (CN), 1706 (C=O), 1617(C=N). ^1^H-NMR (CDCl_3_) δ:2.23 (s, 3H, CH_3_), 7.25–7.29 (t, 1H, *J* = 8 Hz, Ar–H), 7.51–7.57 (m, 4H, Ar–H), 7.71–8.01 (m, 9H, Ar–H and NH), and 79.84 (s, br. 1H, NH); ^13^C-NMR (100 MHz) (DMSO-d_6_): δ 14.2(CH_3_), 86.1, 95.4, 115.4, 116.0, 125.8, 127.2, 128.8, 130.1, 130.7, 131.4, 134.6, 137.8, 138.1, 139.3, 145.4, 150.3, 152.7, 164.1, 165.8, 171.2. MS (m/z  %): 530 (M^+^, 0.7), 338 (64), 323 (8), 263 (85), 249 (45), 222 (38), 204 (13), 106 (22), 76 (100), 60 (54), 59 (13). *Anal*. Calcd. For C_29_H_18_N_6_O_3_S (530.56), C, 65.65; H, 3.42; N, 15.84; S, 6.04 **Found**: C, 65.55; H, 3.24; N, 15.65; S, 6.12.

### 2-(2-(1-(4-(1,3-Dioxoisoindolin-2-yl)phenyl)ethylidene)hydrazinyl)-5-oxo-7-(thien-2-yl)-4,5-dihydrothiazolo[4,5-b]pyridine-6-carbonitrile (**16b**)

Beige needles (95%, yield); mp: 265 °C; IR (KBr, cm^−1^): 3265 (NH), 2202 (CN), 1706 (C=O), 1617 (C=N). ^1^H-NMR (CDCl_3_) δ:2.33 (s, 3H, CH_3_), 7.26 (t, 1H, *J* = 8 Hz, thienyl), 7.59 (m, 3H, Ar–H), 7.84 (d, 2H, *J* = 8 Hz, Ar–H), 7.98–8.01 (m, 4H, Ar–H, NH), 8.1 (d, 2H, *J* = 8 Hz, thienyl), 9.48 (s, 1H, NH); ^13^C-NMR (100 MHz) (DMSO-d_6_): δ 14.2 (CH_3_), 86.1, 96.8, 112.4, 116.7, 120.7, 120.7, 125.6, 125.8, 127.0, 128.8, 131.4, 132.6, 134.7, 137.4, 139.5, 143.9, 150.2, 164.0, 166.8, 169.2. MS (m/z  %): 536 (M^+1^, 0.4), 470 (1.2), 368 (2), 365 (0.6), 250 (84), 222 (25), 166 (45), 104 (14), 97 (15), 71 (25), 62 (16), 55 (20). *Anal* Calcd. for C_27_H_16_N_6_O_3_S_2_ (536.58) C, 60.44; H, 3.01; N, 15.66; S, 11.95. Found: C, 60.32; H, 3.11; N, 15.55; S, 12.10.

### Alternative synthesis of (16a) and (16b)

In acetic acid (25 mL), equimolar amounts of (**15a**) or (**15b**), ammonium acetate, and arylidenemalonitrile were heated for 4 h under reflux. The reaction mixture was left at room temperature to cool. The solid formed was filtered off, dried and recrystallized from methanol to obtain an identical product with (**16a**) and (**16b**) in all aspects of mp, mixed mp, and spectra.

### Evaluation of the antitumor activity using viability assay

The carcinoma cell line utilized during this study, MCF-7, was gotten from the American Kind Culture Group (ATCC, Minnesota, USA). RPMI-1640 environment was utilized for culturing and keeping of the human tumor cell lines [30]. The medium was supplied in a powder form. 10.4 Gram powder and 2 g NaHCO_3_ in 1 L distilled H_2_O are dissolved to prepare the working solution. Then, in the Melibor bacteria filter (0.22 μm), the medium was sterilized by filtration. The refrigerator was used at 0–4 °C to maintain the prepared medium. The medium was heated at 37 °C in a water bath and supplemented with 1% penicillin streptomycin and 10% of fetal bovine serum before use. Through the use of the sulforhodamine-B (SRB) assay [31] the cytotoxicity assay was performed. SRB is aminoxanthrene dye with two SO_3_H groups. It is a protein patches that binds to the amino groups of intracellular proteins under slightly acidic conditions to supply a sensitive indicator of cellular protein content. In MCF-7 cells, cytotoxicity was tested for all compounds. At the National Cancer Institute, Cairo, Egypt, by serial subculturing all experiments and data related to the assessment of cellular cytotoxicity were conducted. For the cytotoxicity assay, cells were seeded in 96-well microliter plates at an initial concentration of 3 × 10^3^ cells/well in 150 µL of fresh medium and left to attach to the plates for 24 h. At variable concentrations of 0, 5, 12.5, 25 and 50 μg/mL, the drug was added. Three wells were used for every concentration of drugs and the plates were incubated for 48 h. The cells were fixed by adding 50 μL of cold trichloro acetic acid (10% final concentration) at 4 °C for 1 h. The plates were therefore washed with distilled H_2_O using an automatic washer (Tecan, Germany) and stained with 50 μL of 0.4%. SRB dissolved in 1% acetic acid at room temperature for 30 min. With 1% acetic acid and dried air, the plates were washed. 100 μL/well of 10 M Tris base (pH 10.5) solubilized the dye. With an ELISA microplate reader (Sunrise Tecan Reader, Tecan, Germany), the optical density (O.D.) of each well was measured spectrophotometrically at 570 nm. The mean absorption of the background was automatically subtracted and the mean values were calculated for each concentration of drugs. Three times the experiment was repeated. The cell survival percentage was calculated as follows: Fraction surviving = O.D. (treated cells)/O.D. (control cells). The values of inhibitory concentration (IC_50_) (resveratrol concentrations required to inhibit cell growth by 50%) have also been calculated. The relationship between the surviving cells and the concentration of the drug was plotted after treatment with the specified compound to obtain the survival curve of each tumor cell line. The IC_50_, The concentration required by 50% of intact cells to cause toxic effects, was estimated at each concentration from graphical plots of the dose response curve.

## Conclusions

Compound **1** was useful for synthesizing a new series of 1,3-thiazole, pyrano[2,3-*d*]thiazole and 4,5-dihydrothiazolo[4,5-*b*]pyridine derivatives using hydrazonoyl halides as precursors. The anticancer effectiveness of compounds **9b**, **9e**, and **9f** against the MCF-7, breast cancer cell line, was also compared with that of the standard anticancer drug doxorubicin.

## Additional files

**Additional file 1: Figure S1.** The ^1^H NMR and ^13^C NMR of compound **(3).**

**Additional file 2: Figure S2.** The ^1^H NMR and ^13^C NMR of compound **(9a).**

**Additional file 3: Figure S3.** The ^1^H NMR and ^13^C NMR of compound **(9b).**

**Additional file 4: Figure S4.** The ^1^H NMR and ^13^C NMR of compound **(9c).**

**Additional file 5: Figure S5.** The ^1^H NMR and ^13^C NMR of compound **(9d).**

**Additional file 6: Figure S6.** The ^1^H NMR and ^13^C NMR of compound **(9e).**

**Additional file 7: Figure S7.** The ^1^H NMR and ^13^C NMR of compound **(9f).**

**Additional file 8: Figure S8.** The ^1^H NMR and ^13^C NMR of compound **(10).**

**Additional file 9: Figure S9.** The ^1^H NMR and ^13^C NMR of compound **(12a).**

**Additional file 10: Figure S10.** The ^1^H NMR and ^13^C NMR of compound **(12b).**

**Additional file 11: Figure S11.** The ^1^H NMR and ^13^C NMR of compound **(12c).**

**Additional file 12: Figure S12.** The ^1^H NMR of compound **(13).**

**Additional file 13: Figure S13.** The ^1^H NMR and ^13^C NMR of compound **(15a).**

**Additional file 14: Figure S14.** The ^1^H NMR and ^13^C NMR of compound **(15b).**

## Abbreviations

MCF-7: the breast cancer cell line
IC_50_: the concentration required to cause toxic effects in 50% of intact cells
O.D.: the optical density
ATCC: the American Type Culture Collection
mp: melting point
Mw: molecular weight

## References

[1] Bharti SK, Nath G, Tilak R, Singh S (2010). Synthesis, anti-bacterial and anti-fungal activities of some novel Schiff bases containing 2, 4-disubstituted thiazole ring. Eur J Med Chem.

[2] Yang BV, Weinstein DS, Doweyko LM, Gong H, Vaccaro W, Huynh T, Xiao H-y, Doweyko AM, Mckay L, Holloway DA (2010). Dimethyl-diphenyl-propanamide derivatives as nonsteroidal dissociated glucocorticoid receptor agonists. J Med Chem.

[3] Spector F, Liang L, Giordano H, Sivaraja M, Peterson M (1998). Inhibition of herpes simplex virus replication by a 2-amino thiazole via interactions with the helicase component of the UL5-UL8-UL52 complex. J Virol.

[4] González Cabrera D, Douelle F, Feng T-S, Nchinda AT, Younis Y, White KL, Wu Q, Ryan E, Burrows JN, Waterson D (2011). Novel orally active antimalarial thiazoles. J Med Chem.

[5] Bell FW, Cantrell AS, Hoegberg M, Jaskunas SR, Johansson NG, Jordan CL, Kinnick MD, Lind P, Morin JM (1995). Phenethylthiazolethiourea (PETT) compounds, a new class of HIV-1 reverse transcriptase inhibitors. 1. Synthesis and basic structure-activity relationship studies of PETT analogs. J Med Chem.

[6] Fink BE, Mortensen DS, Stauffer SR, Aron ZD, Katzenellenbogen JA (1999). Novel structural templates for estrogen-receptor ligands and prospects for combinatorial synthesis of estrogens. Chem Biol.

[7] Biagetti M, Leslie CP, Mazzali A, Seri C, Pizzi DA, Bentley J, Genski T, Di Fabio R, Zonzini L, Caberlotto L (2010). Synthesis and structure–activity relationship of *N*-(3-azabicyclo [3.1. 0] hex-6-ylmethyl)-5-(2-pyridinyl)-1, 3-thiazol-2-amines derivatives as NPY Y5 antagonists. Bioorg Med Chem Lett.

[8] Van Tilburg E, Van der Klein P, De Groote M, Beukers M (2001). IJzerman A: substituted 4-phenyl-2-(phenylcarboxamido)-1, 3-thiazole derivatives as antagonists for the adenosine A1 receptor. Bioorg Med Chem Lett.

[9] Bhoga U (2007). Novel synthetic approach to *N*-aryl-4-(3-pyridyl) thiazol-2-amine and analogues using HMCM-41 as catalyst, and their biological evaluation as human platelet aggregation inhibitors. Eur J Med Chem.

[10] Wilson KJ, Illig CR, Subasinghe N, Hoffman JB, Rudolph MJ, Soll R, Molloy CJ, Bone R, Green D, Randall T (2001). Synthesis of thiophene-2-carboxamidines containing 2-amino-thiazoles and their biological evaluation as urokinase inhibitors. Bioorg Med Chem Lett.

[11] Zhang W-T, Ruan J-L, Wu P-F, Jiang F-C, Zhang LN, Fang W, Chen X-L, Wang Y, Cao B-S, Chen G-Y (2009). Design, synthesis, and cytoprotective effect of 2-aminothiazole analogues as potent poly (ADP-ribose) polymerase-1 inhibitors. J Med Chem.

[12] Carter JS, Kramer S, Talley JJ, Penning T, Collins P, Graneto MJ, Seibert K, Koboldt CM, Masferrer J, Zweifel B (1999). Synthesis and activity of sulfonamide-substituted 4, 5-diaryl thiazoles as selective cyclooxygenase-2 inhibitors. Bioorg Med Chem Lett.

[13] Badorc A, Bordes M-F, de Cointet P, Savi P, Bernat A, Lalé A, Petitou M, Maffrand J-P, Herbert J-M (1997). New orally active non-peptide fibrinogen receptor (GpIIb-IIIa) antagonists: identification of ethyl 3-[*N*-[4-[4-[amino [(ethoxycarbonyl) imino] methyl] phenyl]-1, 3-thiazol-2-yl]-*N*-[1-[(ethoxycarbonyl) methyl] piperid-4-yl] amino] propionate (SR 121787) as a potent and long-acting antithrombotic agent. J Med Chem.

[14] Rudolph J, Theis H, Hanke R, Endermann R, Johannsen L, Geschke F-U (2001). seco-Cyclothialidines: new concise synthesis, inhibitory activity toward bacterial and human DNA topoisomerases, and antibacterial properties. J Med Chem.

[15] Cantin L, Choi S, Clark RB, Hentemann MF, Ma X, Rudolph J. PCT Int. Appl. WO. 2004, 58, 174; Chem. Abstr. 2004, 141, 123483x

[16] Salem MA, Helal M, Gaby M, Ammar Y, Gouda M, Abbas S (2018). Pyrano [2,3-*d*] thiazole: synthesis, reactions and biological applications. Chem J.

[17] Abdelhamid AO, Abdel-Riheem NA, Emam HA (1999). Reactions with hydrazonoyl halides. Part XXV. Synthesis of some new 2, 3-dihydro-1, 3, 4-thiadiazoles and 5-arylazothiazoles. J Chem Res Synopses.

[18] Abdelhamid AO, Elghandour AH, Ahmed SA, Zaki YH (2006). Synthesis and reactions of 2-chloro-2-(hydroximino)-1-(4-methyl-2-phenylthiazol-5-yl) ethanone. J Heterocycl Chem.

[19] Abdelhamid AO, Sayed AR, Zaki YH (2007). Reaction of hydrazonoyl halides 511: a facile synthesis of 5-arylthiazoles and triazolino [4, 3-*a*] pyrimidines as antimicrobial agents. Phosphorus Sulfur Silicon.

[20] Abdelhamid AO, Sayed AR (2007). Reaction of hydrazonoyl halides 52: synthesis and antimicrobial activity of some new pyrazolines and 1, 3, 4-thiadiazolines. Phosphorus Sulfur Silicon.

[21] Abdelhamid AO, Ismail ZH, El Gendy MS, Ghorab MM (2008). Reactions with hydrazonoyl halides 53: 1 synthesis and antimicrobial activity of triazolino [4, 3-a] pyrimidines and 5-arylazothiazoles. Phosphorus Sulfur Silicon Relat Elem.

[22] Popsavin M, Spaić S, Svirčev M, Kojić V, Bogdanović G, Popsavin V (2012). Synthesis and in vitro antitumour screening of 2-(β-d-xylofuranosyl) thiazole-4-carboxamide and two novel tiazofurin analogues with substituted tetrahydrofurodioxol moiety as a sugar mimic. Bioorg Med Chem Lett.

[23] Kaminskyy D, Kryshchyshyn A, Nektegayev I, Vasylenko O, Grellier P, Lesyk R (2014). Isothiocoumarin-3-carboxylic acid derivatives: synthesis, anticancer and antitrypanosomal activity evaluation. Eur J Med Chem.

[24] Eweiss N, Osman A (1980). Synthesis of heterocycles. Part II. New routes to acetylthiadiazolines and alkylazothiazoles. J Heterocycl Chem.

[25] Shawali AS, Abdelhamid AO (1976). Reaction of dimethylphenacylsulfonium bromide with *N*-nitrosoacetarylamides and reactions of the products with nucleophiles. Bull Chem Soc Jpn.

[26] Shawali A, Osman A (1971). Synthesis and reactions of phenylcarbamoylarylhydrazidic chlorides. Tetrahedron.

[27] Abdelhamid AO, El Shiaty FH (1988). Reactions with hydrazidoyl halides II [1]: synthesis and reactions of 2-Bromothienylglyoxal-2-phenylhydrazone. Phosphorus Sulfur Silicon Relat Elem.

[28] Hassaneen H, Shawali A, Elwan N, Abounada N (1992). Reaction of 1-(2-naphthoyl) methyl-2-dimethylsulfonium bromide with *N*-nitroso-*N*-arylacetamides and reactions of the products with some nucleophiles. Sulfur Lett.

[29] Asiri AM, Al-Youbi AO, Zayed ME, Ng SW (2011). 1-Chloro-1-[(4-chlorophenyl) hydrazinylidene] propan-2-one. Acta Crystallogr Sect E Struct Rep Online.

[30] Skehan P, Storeng R, Scudiero D, Monks A, McMahon J, Vistica D, Warren JT, Bokesch H, Kenney S, Boyd MR (1990). New colorimetric cytotoxicity assay for anticancer-drug screening. J Natl Cancer Ins.

[31] Vichai V, Kirtikara K (2006). Sulforhodamine B colorimetric assay for cytotoxicity screening. Nat Protoc.

