# Supplementary figures and images for: Synthesis of novel thiazole, pyranothiazole, thiazolo[4,5-b]pyridines and thiazolo[5′,4′:5,6]pyrano[2,3-d]pyrimidine derivatives and incorporating isoindoline-1,3-dione group
Source: BMC Chem. 2019 Mar 26;13(1):37. doi: 10.1186/s13065-019-0559-x (PMC6661834; doi:10.1186/s13065-019-0559-x)

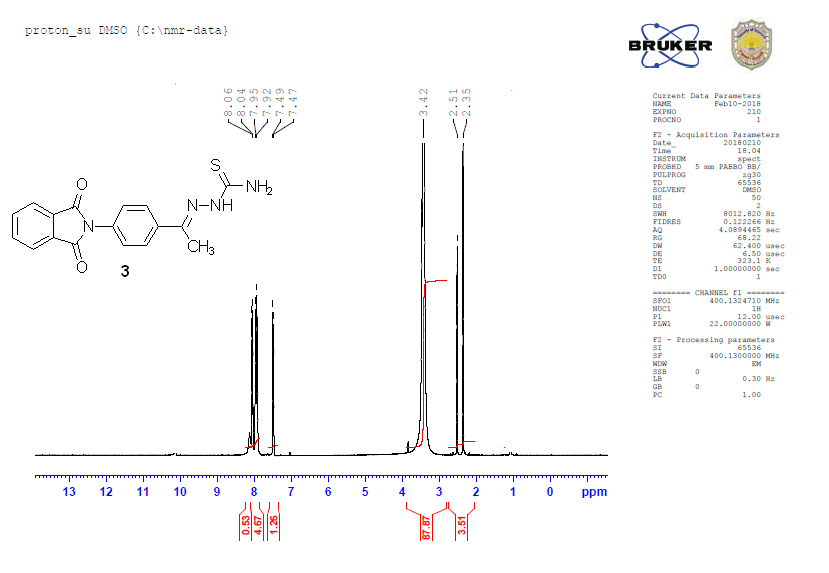


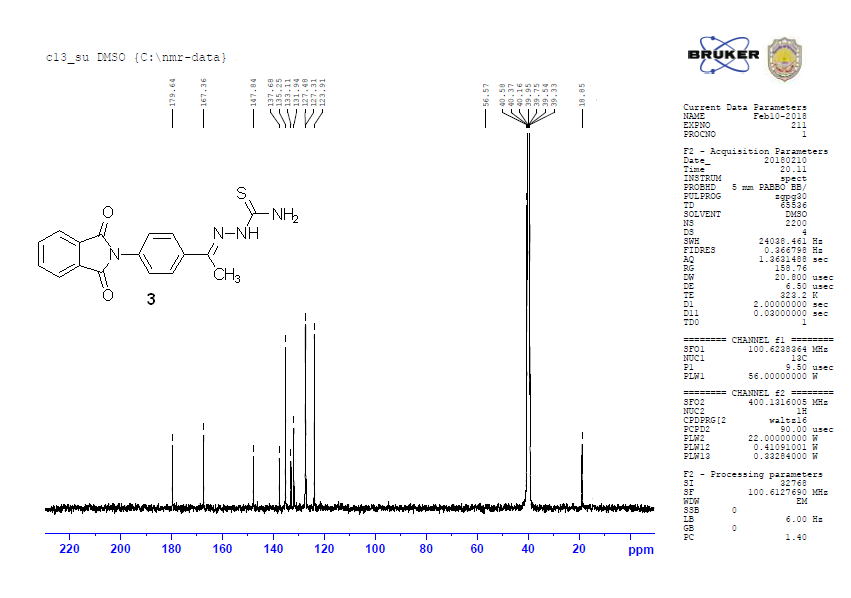


Figure S1: The 1H NMR and 13C NMR of compound **(3).**

Supplement: Supplementary file 1 — Additional file 1: Figure S1. The 1H NMR and 13C NMR of compound (3). [file 13065_2019_559_MOESM1_ESM.docx]

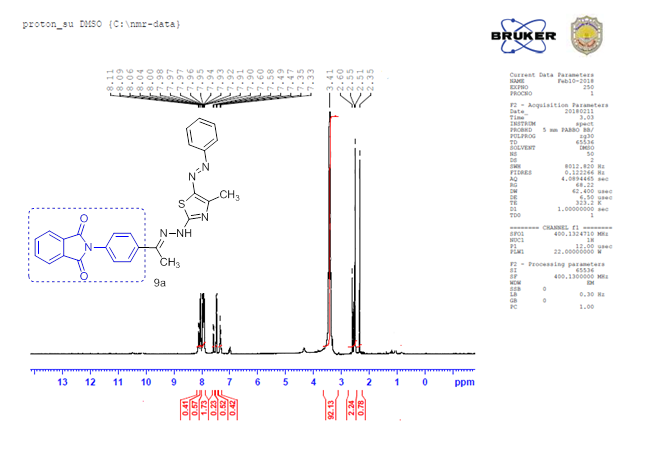


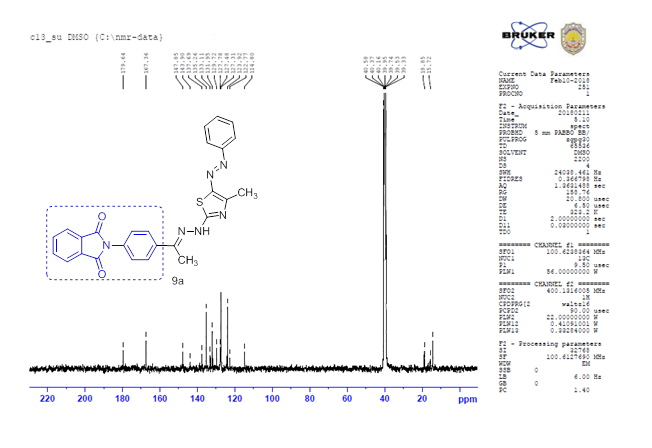


Figure S2: The 1H NMR and 13C NMR of compound **(9a).**

Supplement: Supplementary file 2 — Additional file 2: Figure S2. The 1H NMR and 13C NMR of compound (9a). [file 13065_2019_559_MOESM2_ESM.docx]

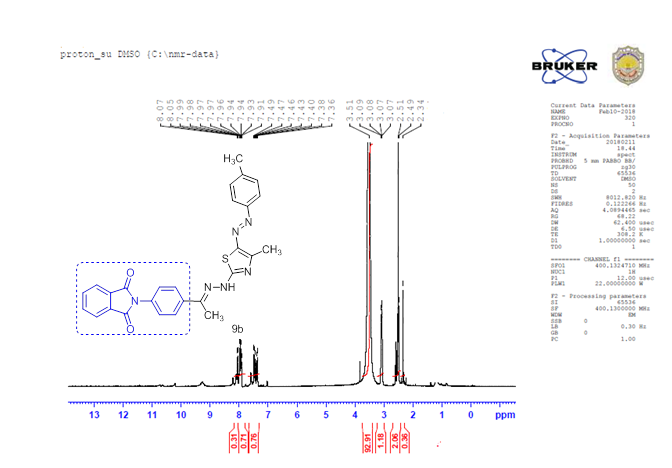


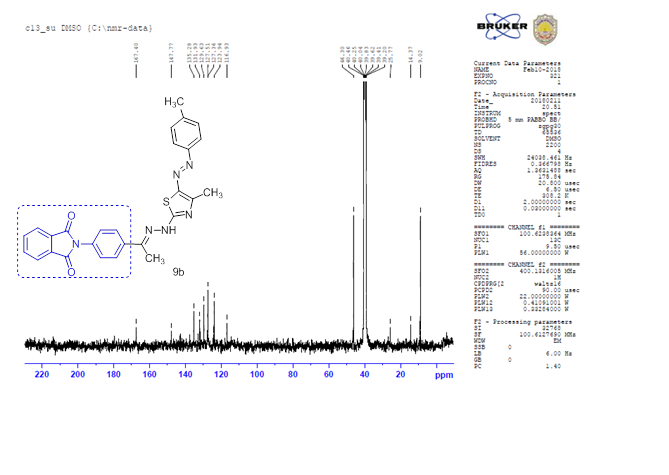


Figure S3: The 1H NMR and 13C NMR of compound **(9b).**

Supplement: Supplementary file 3 — Additional file 3: Figure S3. The 1H NMR and 13C NMR of compound (9b). [file 13065_2019_559_MOESM3_ESM.docx]

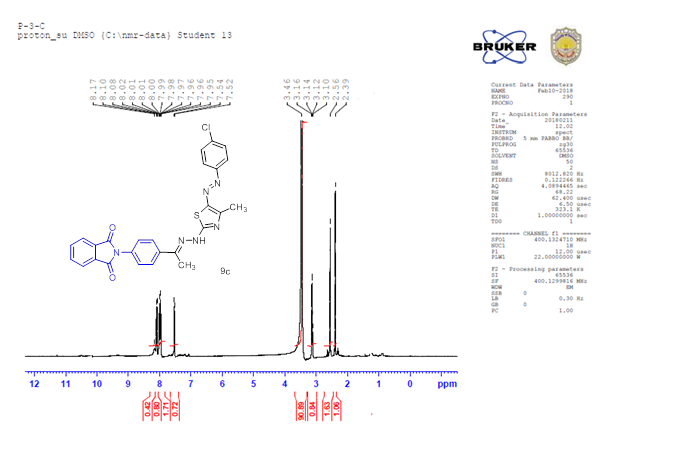


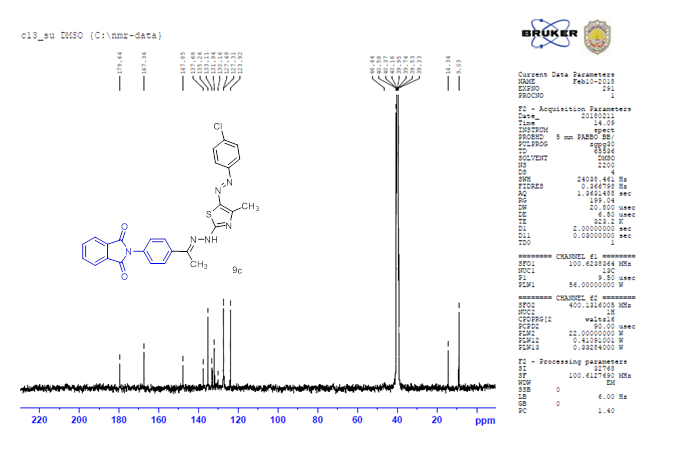


Figure S4: The 1H NMR and 13C NMR of compound **(9c).**

Supplement: Supplementary file 4 — Additional file 4: Figure S4. The 1H NMR and 13C NMR of compound (9c). [file 13065_2019_559_MOESM4_ESM.docx]

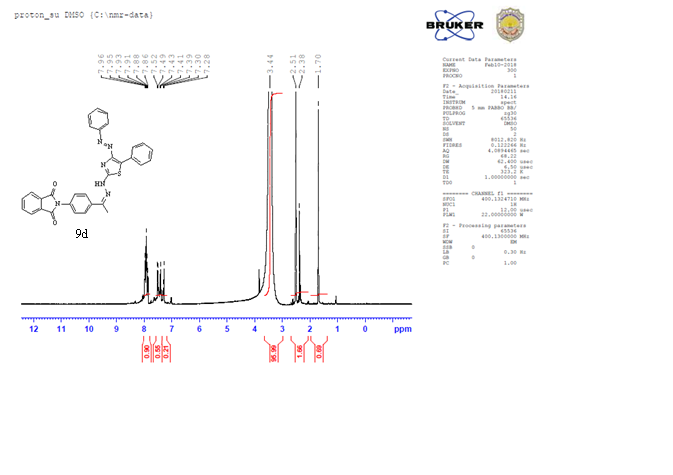


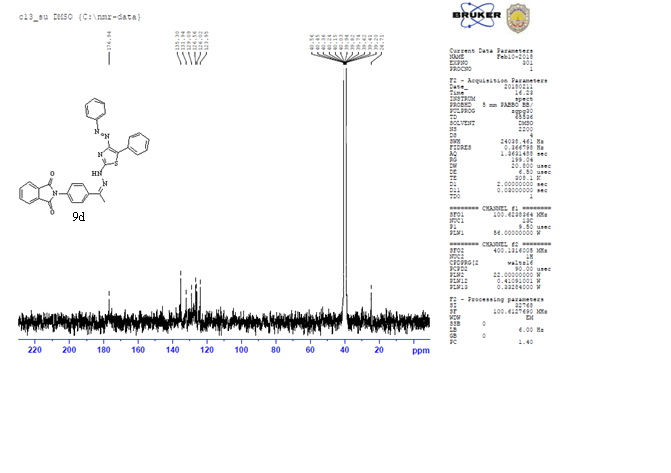


Figure S5: The 1H NMR and 13C NMR of compound **(9d).**

Supplement: Supplementary file 5 — Additional file 5: Figure S5. The 1H NMR and 13C NMR of compound (9d). [file 13065_2019_559_MOESM5_ESM.docx]

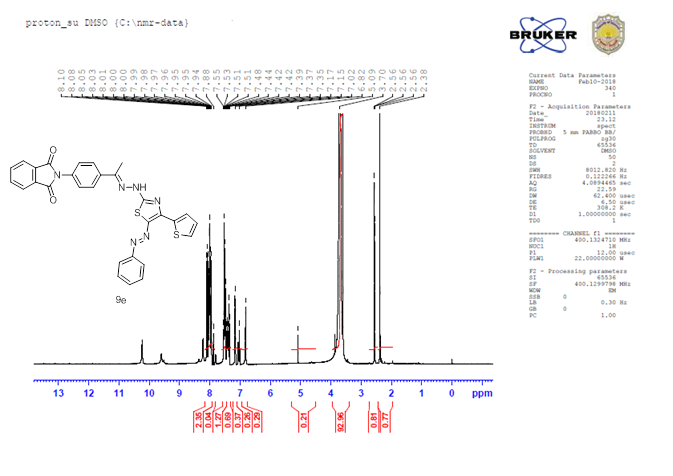


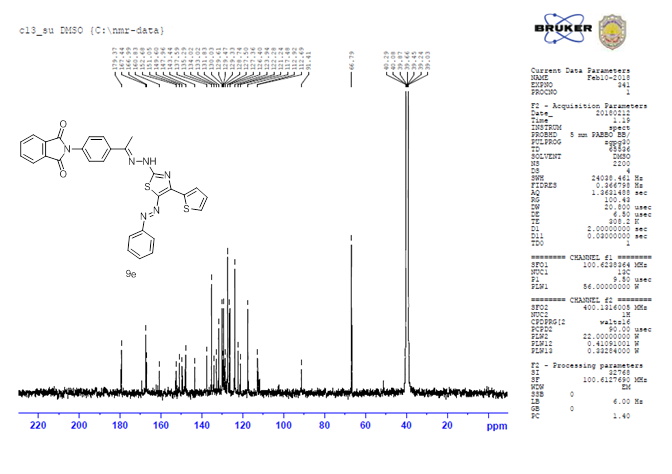
Figure S6: The 1H NMR and 13C NMR of compound **(9e).**

Supplement: Supplementary file 6 — Additional file 6: Figure S6. The 1H NMR and 13C NMR of compound (9e). [file 13065_2019_559_MOESM6_ESM.docx]

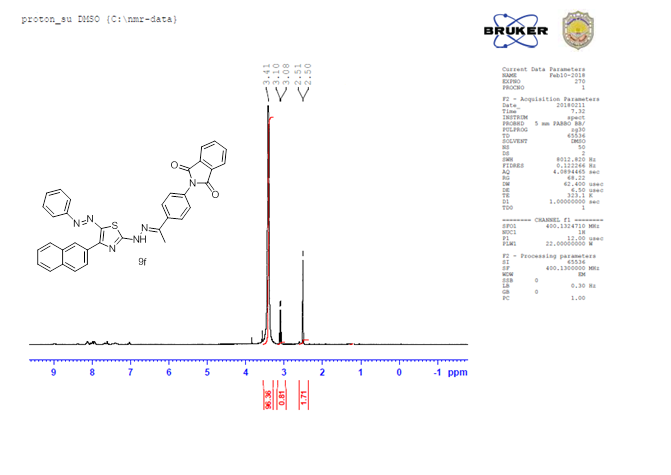

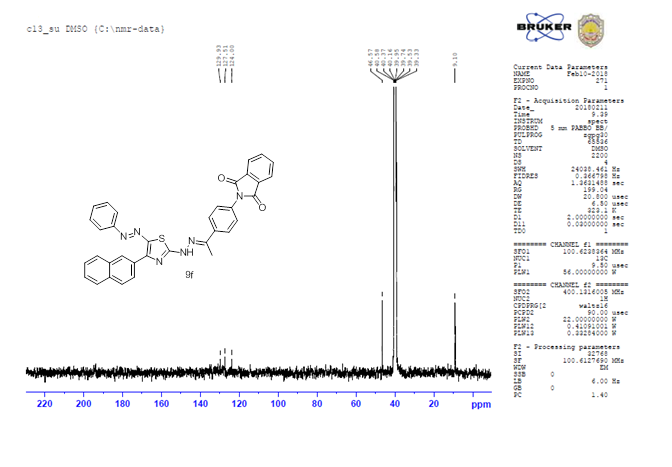


Figure S7: The 1H NMR and 13C NMR of compound **(9f).**

Supplement: Supplementary file 7 — Additional file 7: Figure S7. The 1H NMR and 13C NMR of compound (9f). [file 13065_2019_559_MOESM7_ESM.docx]

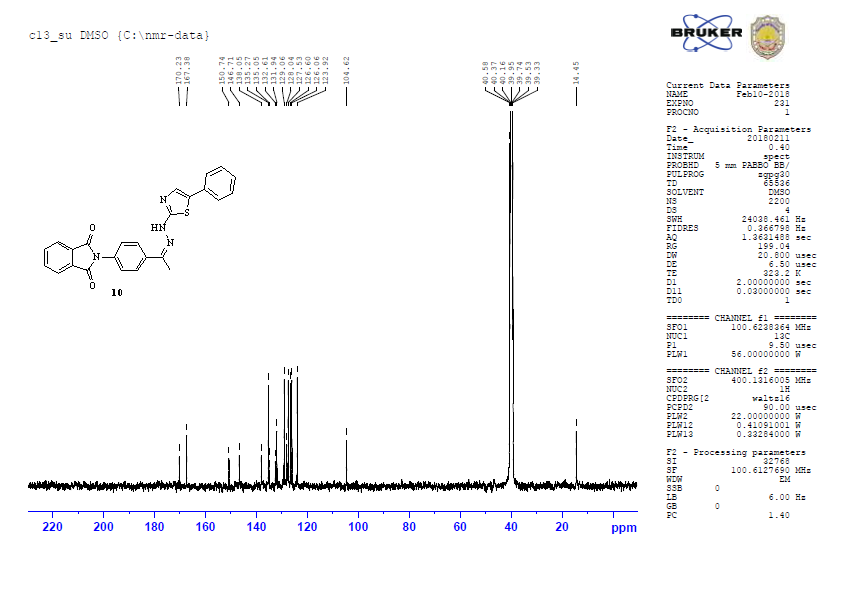

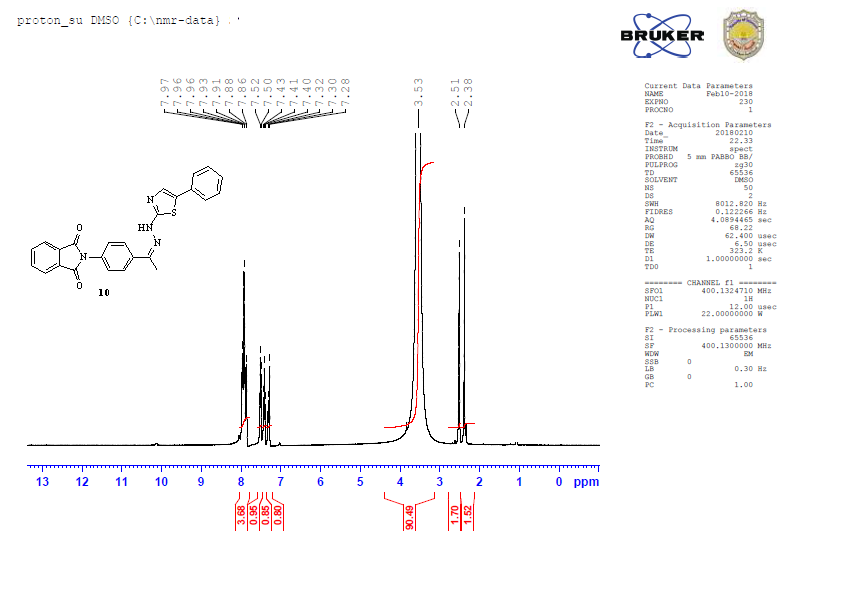


Figure S8: The 1H NMR and 13C NMR of compound **(10).**

Supplement: Supplementary file 8 — Additional file 8: Figure S8. The 1H NMR and 13C NMR of compound (10). [file 13065_2019_559_MOESM8_ESM.docx]

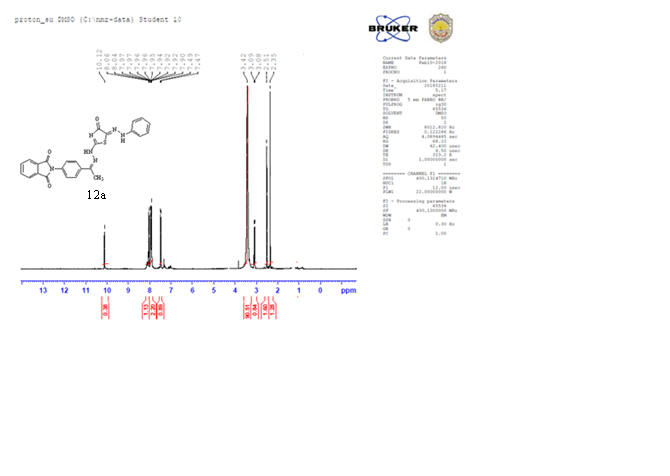


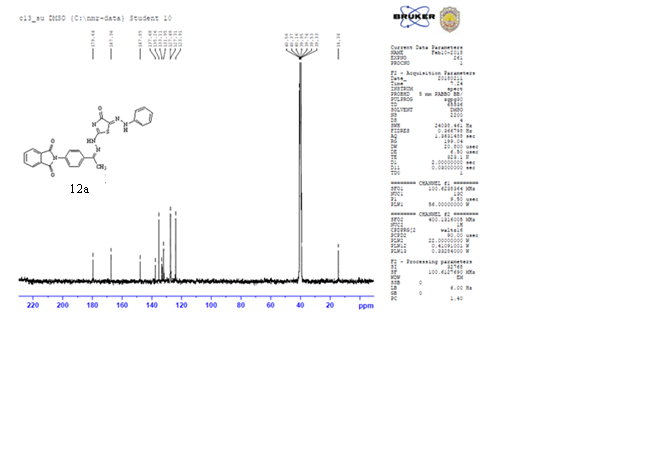


Figure S9: The 1H NMR and 13C NMR of compound **(12a).**

Supplement: Supplementary file 9 — Additional file 9: Figure S9. The 1H NMR and 13C NMR of compound (12a). [file 13065_2019_559_MOESM9_ESM.docx]

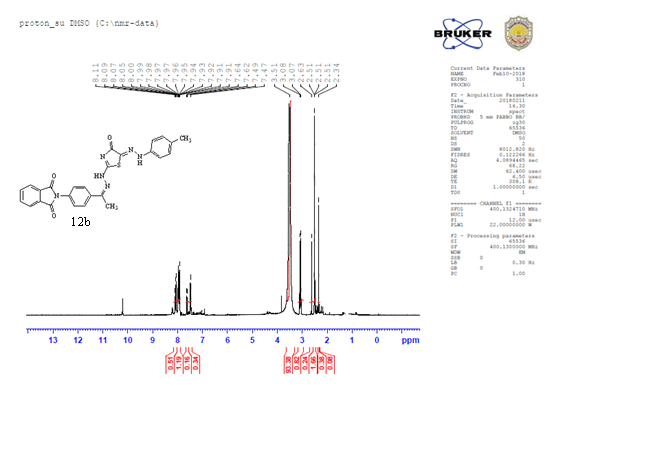


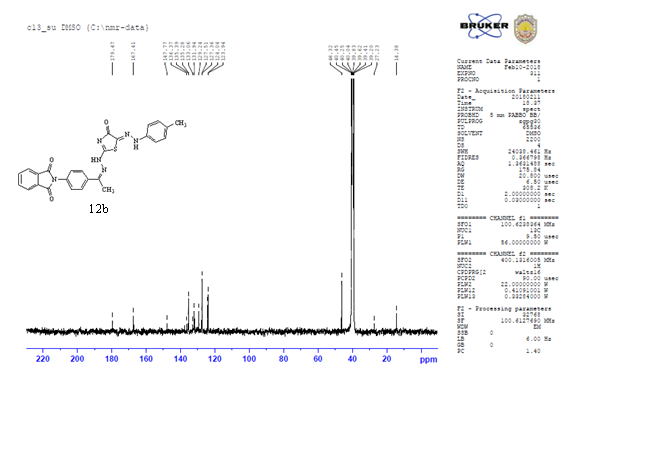
Figure S10: The 1H NMR and 13C NMR of compound **(12b).**

Supplement: Supplementary file 10 — Additional file 10: Figure S10. The 1H NMR and 13C NMR of compound (12b). [file 13065_2019_559_MOESM10_ESM.docx]

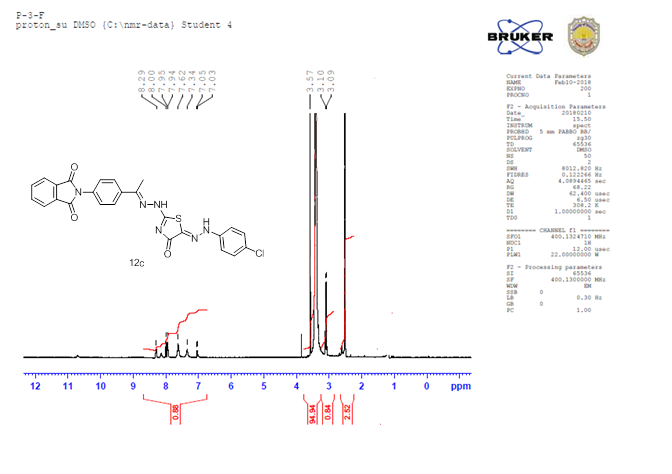

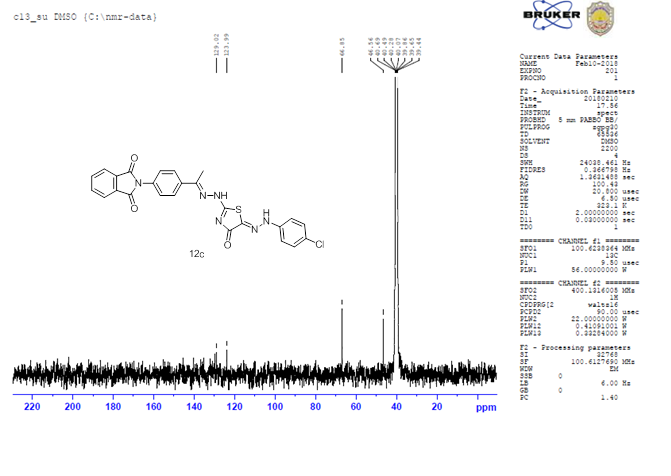


Figure S11: The 1H NMR and 13C NMR of compound **(12c).**

Supplement: Supplementary file 11 — Additional file 11: Figure S11. The 1H NMR and 13C NMR of compound (12c). [file 13065_2019_559_MOESM11_ESM.docx]

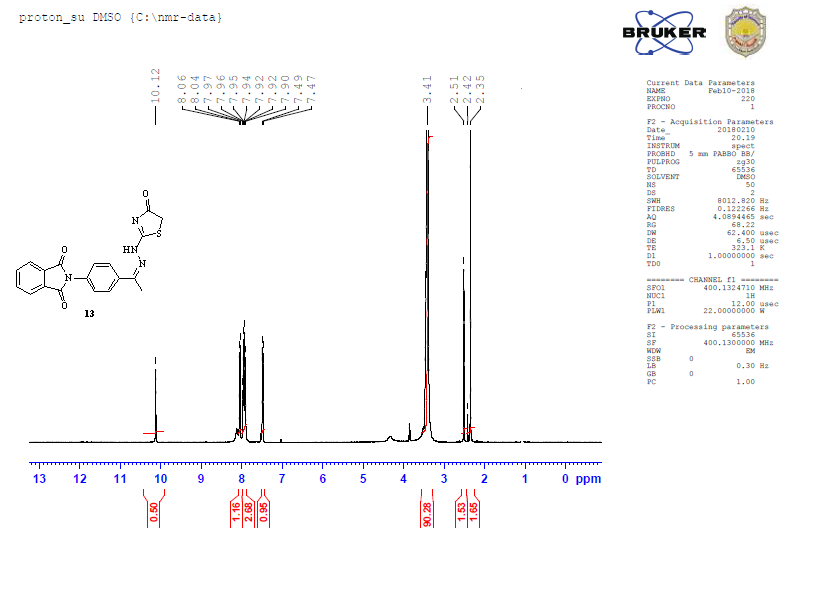


Figure S12: The 1H NMR of compound **(13).**

Supplement: Supplementary file 12 — Additional file 12: Figure S12. The 1H NMR of compound (13). [file 13065_2019_559_MOESM12_ESM.docx]

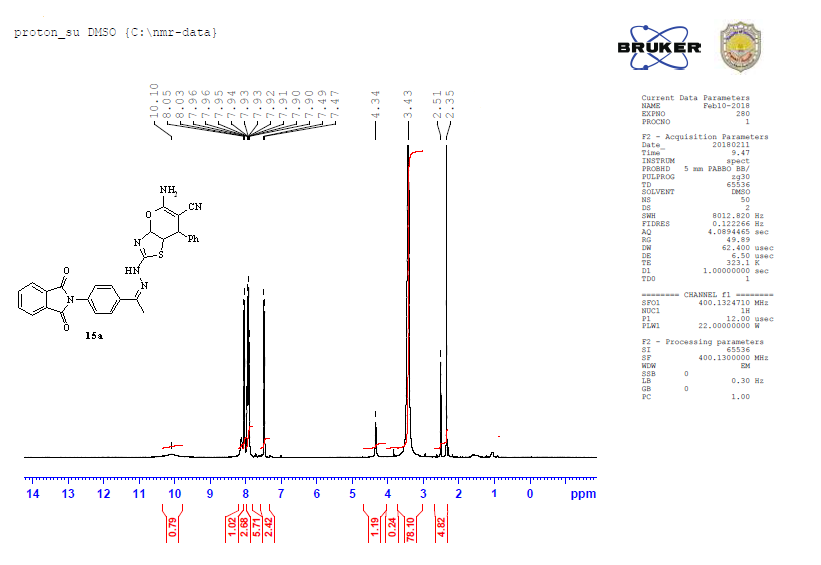


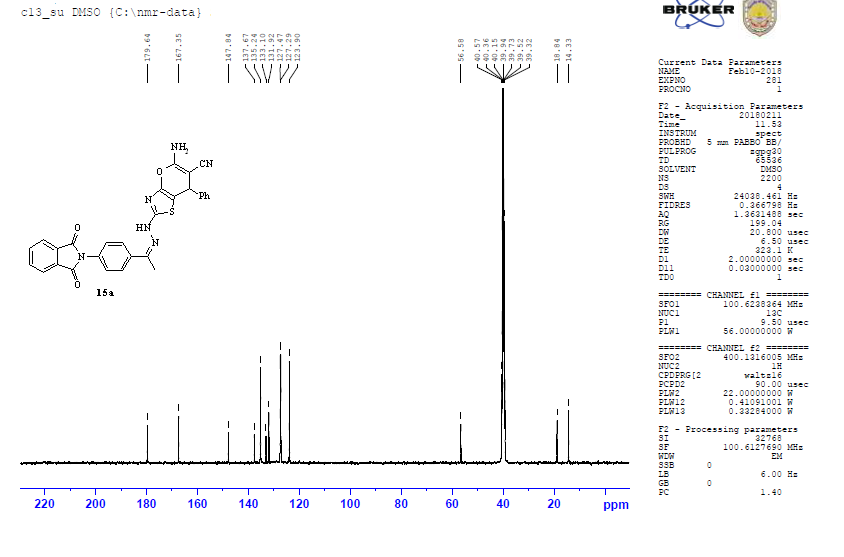


Figure S13: The 1H NMR and 13C NMR of compound **(15a).**

Supplement: Supplementary file 13 — Additional file 13: Figure S13. The 1H NMR and 13C NMR of compound (15a). [file 13065_2019_559_MOESM13_ESM.docx]

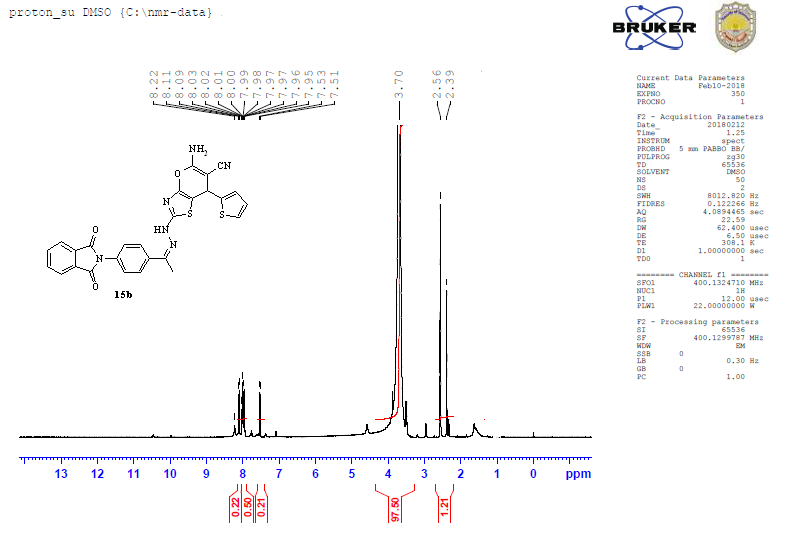

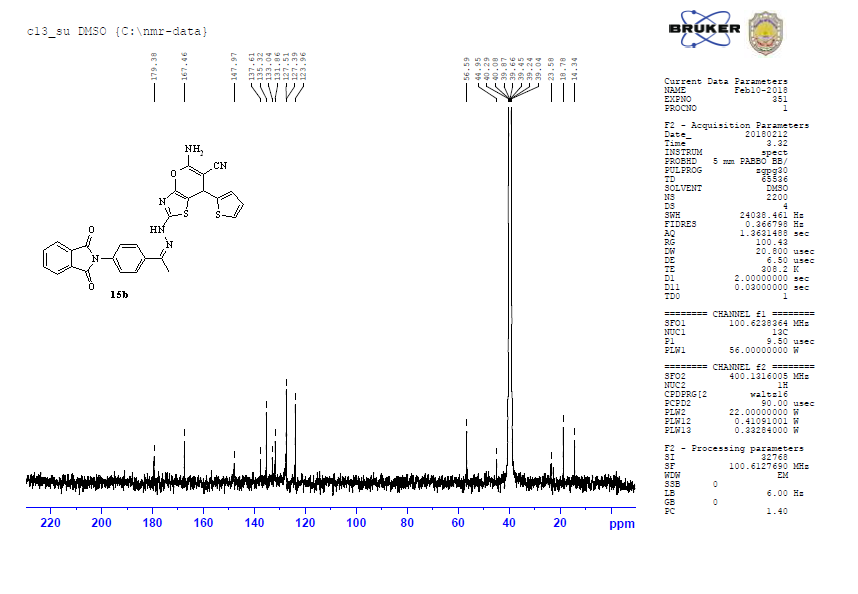


Figure S14: The 1H NMR and 13C NMR of compound **(15b).**

Supplement: Supplementary file 14 — Additional file 14: Figure S14. The 1H NMR and 13C NMR of compound (15b). [file 13065_2019_559_MOESM14_ESM.docx]
